# Cone-beam CT-based synthetic CT generation for adaptive radiotherapy: methodological paradigms, clinical reliability assessment, and translation

**DOI:** 10.3389/fonc.2026.1981244

**Published:** 2026-10-20

**Authors:** Yunxian Zhang, Wanting Zhang, Jinbai Huang, Jingwei Zhao, Huasong Luo, Da He, Zhi Yang

**Affiliations:** 1 First Affiliated Hospital of Yangtze University, Yangtze University, Jingzhou, Hubei, China; 2 Yangtze University Health Science Center, Yangtze University, Jingzhou, Hubei, China; 3 Beijing Jishuitan Hospital, Capital Medical University, Beijing, China; 4 School of Biomedical Engineering, Capital Medical University, Beijing, China; 5 Laboratory for Clinical Medicine, Capital Medical University, Beijing, China

**Keywords:** adaptive radiotherapy, clinical validation, deep learning, medical image synthesis, quantitative imaging

## Abstract

Synthetic computed tomography (sCT) generated from cone-beam computed tomography (CBCT) has important applications in quantitative imaging, dose recalculation, and adaptive radiotherapy. Deep learning has improved image quality and Hounsfield unit accuracy, but its reliability for patient-specific dosimetry and routine clinical use remains uncertain. This narrative review examines deep learning methods for generating sCT from treatment CBCT in photon and proton radiotherapy, with emphasis on data requirements, generalizability, structural fidelity, dosimetric accuracy, and clinical feasibility. The literature search was last updated on 30 July 2026. Current methods mainly include supervised regression, adversarial networks, registrationguided approaches, and diffusion models. Available evidence demonstrates encouraging image and dosimetric performance, although no method consistently performs best across anatomical sites, imaging systems, and treatment modalities. Most studies are based on proof of concept or single-center validation and show considerable heterogeneity in datasets, acquisition protocols, reference images, registration methods, and evaluation criteria. External validation, prospective assessment, failure detection, and quality assurance remain limited. Future studies should emphasize multicenter validation, standardized and modality-specific evaluation criteria, uncertainty assessment, and prospective workflow testing. More rigorous and clinically relevant evidence is needed to support the reliable use of sCT in patient-specific adaptive radiotherapy.

## 1 Introduction

Cone-beam computed tomography (CBCT), when integrated into medical linear accelerator systems, provides fraction-specific anatomical imaging and serves as an important online imaging modality for adaptive radiotherapy (ART) (1, 2). However, scatter, beam hardening, noise, insufficient soft-tissue contrast, and other imaging artifacts compromise the stability and quantitative accuracy of Hounsfield unit (HU) values in CBCT, limiting its direct use for electron density estimation and accurate dose calculation (3, 4). To address this limitation, synthetic CT generation from treatment-fraction CBCT has emerged as an important research direction in ART (5). This task maps treatment-fraction CBCT to synthetic computed tomography (sCT) images with CT-equivalent HU distributions while aiming to correct intensity bias, suppress imaging artifacts, and preserve fraction-specific anatomy (6). Its purpose therefore extends beyond visual enhancement to the recovery of quantitative information required for electron density estimation, dose recalculation, treatment plan evaluation, and adaptive treatment decision-making (5, 6).

Early approaches to CBCT-based dose recalculation included CT value calibration, tissue-density override, deformable registration-based propagation of planning CT, and scatter and intensity correction (7–9). For organizational purposes, deep learning methods for sCT generation from CBCT are discussed in four methodological categories: supervised deterministic regression, adversarial image translation, registrationaware generation, and probabilistic diffusion-based generation (10–13). These categories are not mutually exclusive and describe the dominant methodological characteristics of the reviewed approaches. Supervised regression commonly uses convolutional neural network (CNN) architectures, particularly U-Net and its variants. Adversarial image translation is represented by methods such as Pix2Pix and CycleGAN. Registration-aware models, including RegGAN, seek to mitigate spatial misalignment in the training data through joint registration or registration constraints. Denoising diffusion probabilistic models (DDPMs) model conditional image distributions and generate sCT images through iterative denoising. Multi-domain generative approaches have also been explored for sCT generation from CBCT (14). Hybrid models are discussed according to their primary image-generation mechanism or defining methodological contribution, while auxiliary registration, adversarial, structural, or conditioning components are treated as cross-cutting design features.

These methodological categories entail different trade-offs in data requirements, structural preservation, generation quality, and computational efficiency. Supervised regression models generally provide stable training and efficient inference but depend on high-quality paired data and may produce overly smoothed images (6, 10). Adversarial models may improve image texture and local detail but can introduce structural distortions, hallucinated features, and training instability (14, 15). Registration-aware methods may reduce spatial mismatch between CBCT and reference CT, although their performance depends on registration accuracy and the underlying deformation model (16, 17). Diffusion models show potential for detail recovery and generation stability, but iterative sampling increases inference time and computational cost (13, 15). Accordingly, the field has expanded from image-quality optimization alone to the joint consideration of HU accuracy, anatomical preservation, dosimetric consistency, and workflow efficiency. However, the maturity of the supporting clinical evidence remains uneven across methods (5, 18).

Early reviews broadly covered sCT generation from magnetic resonance imaging (MRI), CBCT, and positron emission tomography (PET), emphasizing network architectures, image-quality metrics, and clinical applications (19). Subsequent systematic reviews focused on CBCT enhancement and sCT generation in ART, examining image-domain generation, projection-domain correction, and dosimetric evaluation (20). Broader radiotherapy reviews situated sCT generation from CBCT within a unified framework encompassing medical image synthesis from MRI, low-dose CT, diagnostic CT, and PET (21, 22). More recent reviews have addressed sCT generation from CBCT, conditional diffusion models, quantitative performance evaluation, dosimetric validation, and clinical workflows (23, 24).

Despite this progress, the available evidence differs substantially in its relevance to clinical use. Improvements in image similarity do not by themselves establish patient-specific structural reliability, dosimetric validity, generalizability, or workflow feasibility (9, 10, 18). Recent reviews have described model architectures, quantitative performance, dosimetric evaluation, and clinical workflows, but these forms of evidence are not equivalent in the conclusions they support. A clinically oriented synthesis therefore requires explicit distinction between methodological feasibility, image and structural performance, task-specific dosimetric validation, and evidence obtained under independent or workflow-relevant conditions.

This review focuses on deep learning methods that generate CT-equivalent sCT from treatment-fraction CBCT for radiotherapy (9, 18, 25, 26). Related studies with different output targets or validation purposes are considered only when they provide relevant methodological or translational context. The evidence is discussed within the same four non-mutually exclusive methodological categories: supervised deterministic regression, adversarial image translation, registration-aware generation, and probabilistic diffusion-based generation. Rather than re-cataloguing model architectures or reported image metrics, the review examines how data conditions and validation designs affect the strength of evidence for structural reliability, dosimetric validity, generalizability, and deployment feasibility. It further distinguishes methodological feasibility from evidence supporting dose recalculation, treatment plan evaluation, and adaptive treatment decision-making (**Figure 1**).

**Figure 1:**
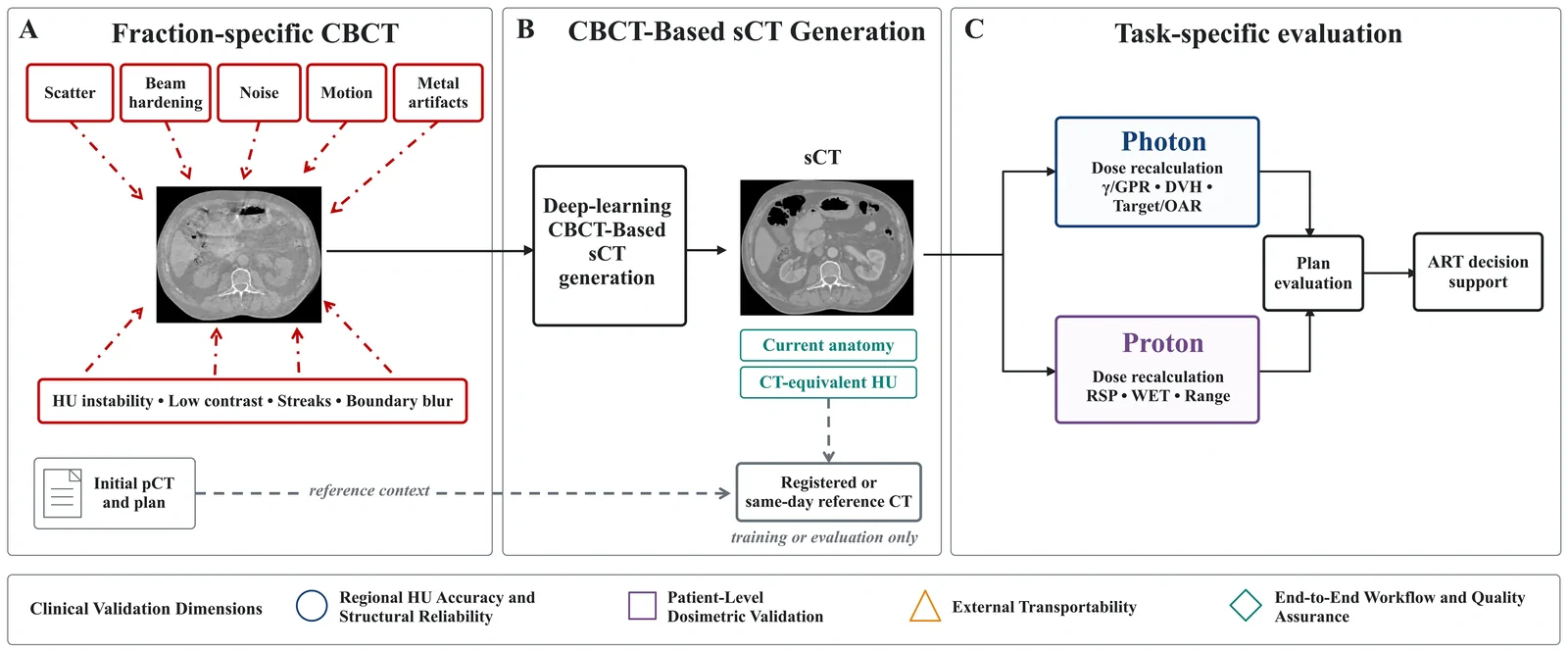
Overview of fraction-specific CBCT-based synthetic CT (sCT) generation and task-specific evaluation for adaptive radiotherapy. **(A)** illustrates how scatter, beam hardening, noise, motion, and metal-related artifacts in CBCT can cause HU instability, reduced tissue contrast, and local structural errors, potentially affecting electron density estimation and subsequent dose evaluation. **(B)** depicts sCT generation from CBCT, which aims to recover CT-equivalent HU distributions while preserving fraction-specific anatomy. **(C)** presents task-specific evaluation, including photon and proton dose recalculation, plan evaluation, and adaptive radiotherapy decision support. Clinical validation encompasses regional HU accuracy, structural reliability, patient-level dosimetric validation, external transportability, and end-to-end workflow quality assurance.

### 1.1 Review scope and literature search

This narrative review used a structured PubMed search, last updated on 30 July 2026, supplemented by reference-list screening and targeted searches for relevant methodological and translational studies. Search terms covered CBCT, synthetic CT, deep learning or generative models, radiotherapy or adaptive radiotherapy, and dose calculation. Studies directly evaluating CT-equivalent sCT generated from treatment CBCT constituted the primary evidence base. Studies unrelated to radiotherapy generation of CT-equivalent synthetic CT from treatment-fraction CBCT generation and studies without relevance to the methodological, quantitative, dosimetric, or translational questions addressed in this review were not included in the evidence synthesis. Dynamic-phantom and workflow studies were treated as preclinical or translational evidence, whereas studies with different output targets or broader image-translation objectives were used only as methodological background. Image-only studies were not interpreted as evidence of dosimetric validity or clinical reliability. Formal risk-of-bias assessment and quantitative evidence synthesis were not performed.

## 2 Synthetic CT generation from CBCT: imaging limitations and clinical requirements

### 2.1 CBCT imaging physics and sources of artifacts

CBCT uses a cone-shaped X-ray beam and a two-dimensional flat-panel detector to acquire projections from multiple angles for three-dimensional volumetric reconstruction (27). Artifacts may arise from X-ray scatter, metal implants, and patient motion, resulting in intensity shifts, reduced contrast, streaking, local information loss, blurred boundaries, and structural misalignment (28, 29). These effects can compromise HU accuracy and anatomical representation, thereby posing major challenges to sCT generation from CBCT.

### 2.2 HU instability and its dosimetric consequences

The limitations of CBCT extend beyond reduced visual image quality. HU instability may compromise electron density estimation and propagate into dose recalculation (28). Consequently, CBCT images adequate for patient positioning or anatomical assessment do not necessarily provide the quantitative reliability required for dosimetric applications (18). sCT generation from CBCT should therefore aim to correct artifacts and intensity bias while recovering HU distributions and electron density information suitable for dose calculation (13).

### 2.3 Task definition and clinical requirements

Medical image synthesis learns mappings from source images to images representing a target modality (30). In ART, however, sCT generation from CBCT is not merely an appearance transformation from CBCT to pCT. Rather, it uses CBCT acquired during the current treatment fraction to generate an sCT image that preserves fraction-specific anatomy while providing a CT-equivalent HU distribution suitable for dose calculation (31). Accordingly, this task should satisfy four principal requirements: patient-specific structural reliability, HU correction and tissue-contrast restoration, dosimetric validity, and deployment feasibility within the intended online or near-online workflow.

## 3 Synthetic CT generation from CBCT: methodological categories and cross-method characteristics

Methods for sCT generation from CBCT differ across several partially overlapping methodological dimensions, including the image-generation mechanism, data pairing, and the treatment of spatial misalignment. Supervised regression, adversarial generation, and diffusion-based generation primarily differ in their learning or generative mechanisms, whereas registration-aware approaches emphasize explicit modeling of CBCT–CT misalignment and may be incorporated into different generative frameworks. Models combining multiple characteristics are discussed according to the component that defines their main synthesis strategy; methods centered on joint registration generation or explicit deformation modeling are discussed as registration-aware approaches. Architectural modules and auxiliary loss constraints are compared across these categories where relevant.

### 3.1 Supervised regression

Supervised regression learns CT-equivalent intensity mappings from paired CBCT–pCT data and was among the earliest deep learning paradigms applied to sCT generation from CBCT. These methods followed earlier quantitative correction strategies, including CT value calibration, density override, deformable propagation, and scatter correction (7–9).

#### 3.1.1 Supervised mapping with CNNs and U-Net

Supervised deep convolutional neural networks (DCNNs) typically learn a direct mapping from CBCT to sCT by minimizing voxel-wise or feature-level differences between the generated sCT and paired pCT or deformed pCT (10, 32). Studies in nasopharyngeal cancer and other ART settings have reported improved HU consistency relative to uncorrected CBCT and evaluated the resulting images using clinical treatment plans (33, 34). These models generally provide stable optimization and rapid inference when sufficiently aligned paired data are available.

U-Net uses an encoder–decoder architecture with skip connections to integrate multiscale contextual information with local spatial detail (35). Existing studies have introduced CT value constraints and contextual or feature-driven losses to improve artifact suppression, tissue-boundary preservation, and HU recovery (32, 36–38). However, voxel-wise regression may average multiple plausible local solutions and produce overly smoothed outputs, whereas skip connections may transmit input artifacts to the generated image. Comparisons across training cohorts further indicate that dosimetric performance depends not only on network architecture but also on paired-data composition, reference-image quality, and anatomical coverage (37, 38).

A major limitation of supervised regression is residual misalignment between CBCT and the reference CT. Interfractional anatomical changes, differences in patient positioning, and variations in organ filling may be incorporated into the learned mapping as apparent intensity differences, thereby affecting boundary preservation and HU estimation (16, 26). Contextual and feature-driven losses may reduce sensitivity to local misalignment but cannot eliminate errors originating from imperfectly registered reference images (32, 38).

#### 3.1.2 Transformers and global context modeling

Transformers and vision transformers (ViTs) model long-range spatial relationships and volumetric context through an expanded effective receptive field. A head-and-neck proton therapy study using a three-dimensional ViT generator reported image-quality and dosimetric results, supporting the feasibility of global-context modeling for paired sCT generation under the evaluated conditions (39). However, volumetric attention increases data, memory, and computational requirements, while performance remains dependent on the registration quality of the reference images.

Overall, supervised regression offers comparatively stable training and efficient inference but depends on high-quality paired data. Residual registration errors, limited cohort coverage, and oversmoothing associated with voxel-wise losses remain important constraints on structural reliability and cross-domain applicability.

### 3.2 Adversarial generation

Adversarial generation uses a discriminator to encourage generated images to follow the target CT-domain distribution. This approach may reduce the oversmoothing associated with direct regression and improve local texture, but it also introduces risks related to optimization instability and non-patient-specific image features (10, 40). The main implementations include paired Pix2Pix and unpaired CycleGAN frameworks.

#### 3.2.1 Pix2Pix and CycleGAN

Pix2Pix combines conditional adversarial learning with a paired reconstruction loss and is therefore suited to settings with relatively close CBCT–pCT correspondence (34, 41, 42). Paired reconstruction provides direct geometric supervision, although model performance remains sensitive to registration accuracy and reference-image quality.

CycleGAN enables unpaired image translation through cycle-consistency constraints and can therefore use clinical CBCT and CT data without strict spatial correspondence (11, 43). This strategy reduces dependence on paired data but lacks direct voxel-wise supervision; consequently, CT-like appearance may be accompanied by local structural deformation or hallucinated features (40, 44). The principal distinction between Pix2Pix and CycleGAN therefore concerns the required degree of data correspondence and the strength of direct geometric constraints.

#### 3.2.2 Volumetric and structure-constrained extensions

To improve interslice continuity and structural preservation, studies have incorporated three-dimensional context, attention mechanisms, Transformer modules, and gradient-, perceptual-, contrastive-, or frequencybased constraints into CycleGAN-derived frameworks (31, 45–50). Three-dimensional and 2.5D inputs incorporate information from adjacent slices, attention and Transformer modules model longerrange dependencies, and specialized loss terms emphasize boundaries, frequencies, or local structural consistency.

Studies across different anatomical sites have reported improvements in image-quality metrics, HU consistency, and selected dosimetric endpoints. However, adversarial distribution matching does not ensure that all generated structures remain patient-specific. Evaluation should therefore include regional HU accuracy, anatomical-boundary preservation, case-level failure analysis, and relevant dosimetric endpoints rather than relying solely on global image metrics.

Overall, adversarial generation reduces dependence on strictly paired data and may improve texture and local detail, but optimization instability, structural drift, and hallucinated features remain important limitations for quantitative and dosimetric applications.

### 3.3 Registration-aware generation

Registration-aware approaches target weakly paired settings in which CBCT and pCT correspond at the patient level but remain insufficiently aligned spatially (16). RegGAN treats label misalignment as learnable noise and incorporates a registration network into the generative framework to jointly optimize image translation and local alignment (17, 51). This formulation makes residual spatial misalignment an explicit component of model optimization rather than an unmodeled perturbation of the generator.

Using head-and-neck data, Chen et al. reported that RegGAN improved HU, structural, and dosimetric endpoints relative to corresponding generators without registration modeling under the conditions of that study (52). Another study reported relatively stable internal cross-device performance using CBCT acquired from different linear accelerators within the same center (12). Thoracic and esophageal studies also reported favorable three-dimensional continuity and preservation of small anatomical boundaries (53).

These findings suggest that some synthesis errors may reflect unmodeled registration noise rather than insufficient generator capacity alone (16, 52). Nevertheless, registration-aware generation assumes that the deformation field can be adequately modeled, that the associated noise distribution is learnable, and that the training data represent the anatomical variations encountered in clinical practice (16, 17). Learned deformation fields may introduce additional bias, and adversarial optimization may still produce non-patient-specific structural alterations (40, 49, 54).

Registration-aware approaches therefore improve the handling of weakly paired clinical data, but their structural reliability and dosimetric validity require further evaluation using independent datasets, regionalerror analyses, and case-level uncertainty estimates (18, 55).

### 3.4 Diffusion-based generation

Diffusion models learn a forward noising process and a CBCT-conditioned reverse denoising process to model the distribution of target sCT images (13, 56, 57). Unlike generator–discriminator frameworks, diffusion models do not rely on minimax optimization and generate images through progressive denoising. This formulation may reduce instability and mode collapse associated with adversarial training (15, 57).

#### 3.4.1 Conditional diffusion models

A conditional denoising diffusion probabilistic model (cDDPM) progressively reconstructs sCT under CBCT guidance. Using paired brain and head-and-neck datasets, Peng et al. reported better image metrics and visual results for cDDPM than for a supervised CycleGAN comparator evaluated within the same study under identical registration and evaluation conditions (13). These results support the technical feasibility of conditional diffusion under the evaluated data conditions, although dosimetric and independent external validation were not reported.

Three-dimensional conditional diffusion has also been applied to dual-energy CT generation from headand-neck CBCT (58). Because dual-energy CT synthesis has different output targets and contrast-related endpoints, these results should not be interpreted directly as evidence of dosimetric validity for conventional CT-equivalent sCT.

Pseudo-CBCT conditioning and hybrid U-Net diffusion models seek to preserve global and local information by simulating acquisition degradation or incorporating ViT, information-bottleneck, and dynamic feature-modulation modules (57, 59). Their performance nevertheless remains dependent on the simulated degradation process, paired target images, and preprocessing pipeline (13, 60).

#### 3.4.2 Structure-guided and unpaired diffusion

Unpaired adversarial diffusion and frequency-guided diffusion provide related methodological foundations for medical image translation (61, 62). For diffusion-based sCT generation from CBCT, energy-guided and texture-preserving models further incorporate domain-energy, frequency, boundary, and cross-feature constraints to improve anatomical preservation (15, 63). Studies using thoracic, pancreatic, pelvic, or head datasets have reported improvements in image metrics, but independent dosimetric validation and workflow-level evidence remain limited.

#### 3.4.3 Accuracy and computational efficiency

The principal deployment constraint of diffusion-based generation is iterative sampling. Compared with single-pass supervised models and GANs, repeated denoising-network evaluations increase computational cost and inference latency (13, 57, 60). Reported processing times are expressed per slice, image, volume, or treatment fraction, and studies differ substantially in hardware, image matrix, spatial dimensionality, and number of sampling steps. Direct efficiency comparisons are therefore generally not valid.

Existing diffusion studies provide preliminary internal image-based evidence and limited external image evaluation. However, many rely on registered reference targets, and reporting of dosimetric endpoints and end-to-end processing time remains incomplete (13, 15, 57). Diffusion models show potential for detail recovery and conditional distribution modeling, but their clinical value requires independent validation, dosimetric assessment, and workflow-level testing.

The training frameworks, data conditions, principal strengths, and limitations of these four methodological categories are summarized in **Figure 2**.

**Figure 2:**
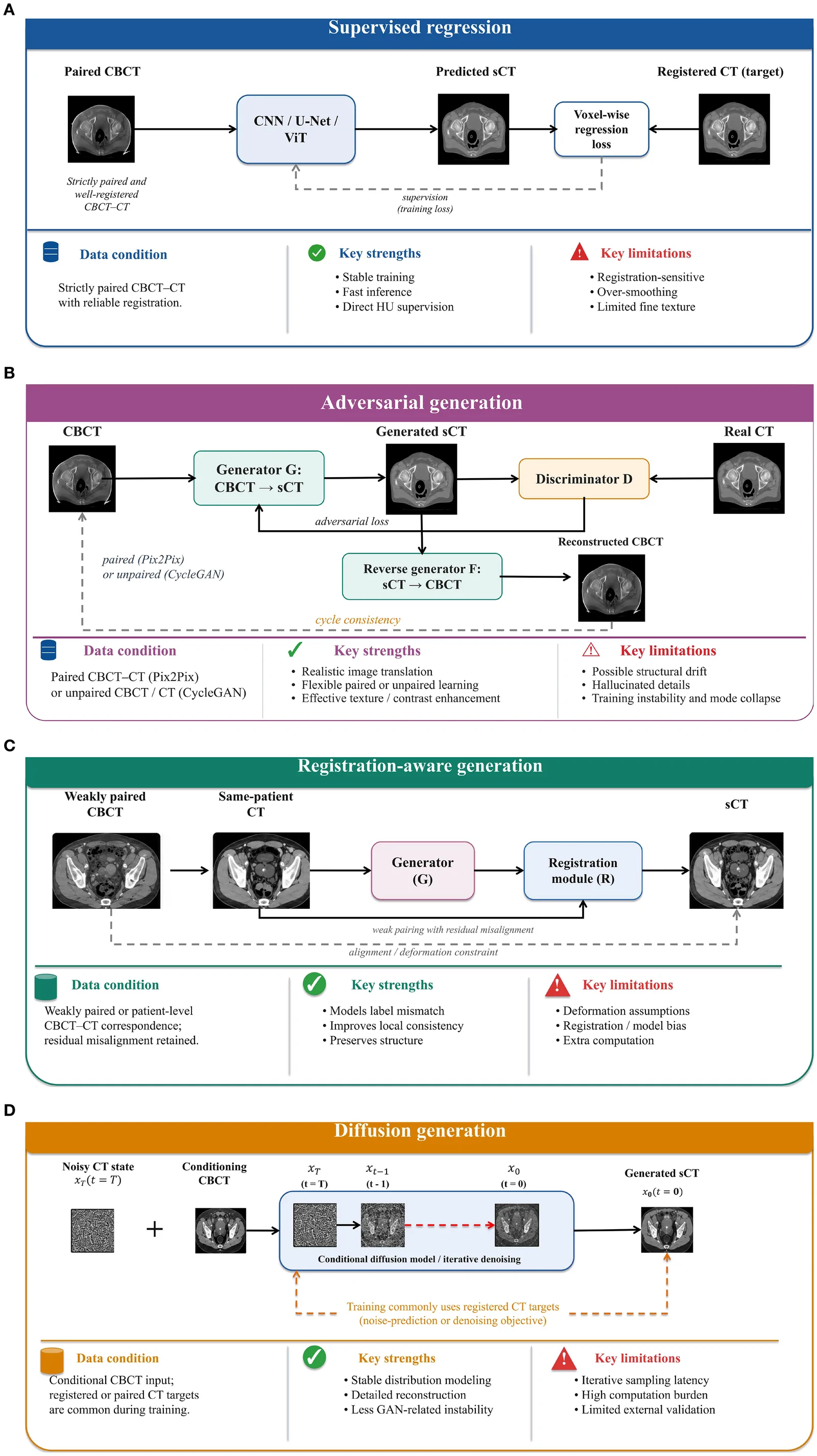
Representative methodological categories for synthetic CT generation from CBCT. **(a)** Supervised regression learns a direct intensity mapping from paired and registered CBCT–CT data. **(b)** Adversarial generation uses paired or unpaired image translation with adversarial and, where applicable, cycle-consistency constraints. **(c)** Registration-aware approaches explicitly model residual spatial misalignment in weakly paired data and may be integrated with different generative frameworks. **(d)** Diffusion-based generation produces sCT through conditional iterative denoising. The categories summarize dominant methodological characteristics and may overlap in hybrid implementations.

### 3.5 Cross-method comparison dimensions

#### 3.5.1 Data pairing, reference images, and registration conditions

Data pairing, reference-image selection, and registration jointly determine the spatial and quantitative correspondence used for model training and evaluation. Planning CT (pCT) refers to the CT acquired for initial treatment planning and may be temporally separated from treatment-fraction CBCT. Same-day repeat CT (reCT) or quality-assurance CT (QACT) provides an independently acquired reference with a shorter temporal interval to CBCT. Registered CT refers to an acquired CT after rigid or deformable spatial alignment, whereas deformed planning CT (dpCT) is generated by deforming the pCT toward the CBCT geometry and is therefore a deformation-derived reference. These reference images differ in anatomical correspondence and dependence on registration and should not be interpreted as equivalent reference standards.

Strictly paired and adequately registered reference images permit voxel-wise HU supervision, but residual misalignment may be incorporated into the learned mapping. Unpaired training reduces dependence on pixel-level correspondence but weakens direct geometric supervision, whereas registration-aware methods explicitly model spatial misalignment or deformation during learning (10, 16, 52).

Differences between sCT and the reference CT may reflect model-related synthesis error, genuine interfractional anatomical change, registration error, or physiological and acquisition-related mismatch. Patient positioning, tumor response, respiratory motion, gas, and organ filling may affect image correspondence (9, 64, 65). For thoracic and abdominal studies, respiratory phase or breath-hold status should therefore be matched or reported where applicable. Bladder and rectal filling and bowel-gas variation should also be considered when relevant to abdominal and pelvic evaluation.

#### 3.5.2 Spatial dimensionality

Two-dimensional models increase the effective number of training samples and reduce memory requirements but do not directly constrain interslice continuity. Three-dimensional models incorporate volumetric context but generally require more computational resources, smaller patches, or smaller batch sizes. In contrast, 2.5D models incorporate adjacent slices to balance computational demand and volumetric information (45, 66, 67).

The appropriate spatial dimensionality depends on anatomical site, motion characteristics, dataset size, and the time constraints of the intended ART workflow. Because structural complexity and motion patterns differ across the head and neck, thorax, and pelvis, methodological performance cannot be inferred solely from whether a model is two- or three-dimensional.

#### 3.5.3 Loss functions and structural constraints

Supervised regression generally uses *L*_1_ or *L*_2_ intensity losses and may incorporate contextual, perceptual, or feature-driven terms to reduce oversmoothing and sensitivity to registration errors (32, 38). Adversarial methods combine target-domain distribution matching with paired reconstruction or cycle consistency and may add contrastive, gradient, frequency, or anatomy-aware losses to strengthen structural constraints (41, 48, 50).

Registration-aware methods additionally constrain deformation fields and alignment processes to reduce the influence of label misalignment on image generation (51, 52). Diffusion methods incorporate frequency-, boundary-, structure-, or energy-guided conditions into denoising or noise-prediction objectives (13, 15, 63). Although these losses may improve selected image properties, their clinical relevance must be established using patient-level structural and dosimetric endpoints.

#### 3.5.4 Computational efficiency and workflow time

Inference time depends on hardware, image matrix, spatial dimensionality, sampling steps, and implementation. Processing times reported per slice, volume, or treatment fraction cannot be directly converted or compared. Moreover, preprocessing, registration, data transfer, dose calculation, and quality assurance (QA) contribute to end-to-end workflow time (17, 60, 68).

Supervised regression and adversarial models generally require a single forward pass and therefore provide relatively efficient image generation. Three-dimensional models impose greater memory and computational demands, registration-aware workflows require additional alignment operations, and diffusion models incur latency from iterative sampling. **Table 1** retains the original time units and hardware information reported by each study and distinguishes model inference time from end-to-end workflow time.

**Table 1:** Methods, data designs, and validation evidence in representative studies of sCT generation from CBCT.

Reference/year	Anatomical site;sample size	Center;scanner/device	Registration/reference image	Training/dataregime	Method;spatial dimension	Validation independence	Dosimetric validation	Inference/workflow time	Image/structural endpoints	Main evidence limitations
Liang et al. (11)	Head and neck; 17 (development, 13; testing, 4)	One center; scanner model NR	Deformation-derived reference: dpCT generated from pCT using Velocity DIR	Unpaired patient domains	CycleGAN; 2D	Internal; single-center	Photon plans, 3D γ; deformable phantom	NR	MAE, RMSE, SSIM, PSNR	Four-patient test; dpCT reference; single-center
Li et al. (33)	Nasopharynx; 70 (50/10/10)	One reported cohort; device NR	CT reference; paired CBCT/CT; registration method NR	Supervised training/validation/test split	DCNN/U-Net; 2D	Internal; one reported cohort	DVH and 3D γ	NR	MAE, ME	Internal single-center test; external and workflow evidence NR
Liu et al. (31)	Pancreas; 30	One center; device NR	CT reference; rigid and deformable registration evaluated	CT domain and first-fraction CBCT domain	Attention CycleGAN; 2D	Internal; single-center	DVH	NR	MAE, SNU, PSNR	Small single-center cohort; no independent test
Zhang et al. (42)	Pelvic development set: 30 patients/150 pairs; test set: 15 pelvic and 10 head-and-neck patients	Number of centers NR; testing included different linear accelerators	CT reference paired with CBCT; registration details NR	Paired data, 10-fold validation plus independent testing	Pix2Pix with feature matching; 2.5D	Cross-device and cross-anatomical-site testing; institutional independence NR	Photon and proton dose recalculation	11–12 ms/slice; < 1 s/80 slices (Titan X)	MAE, PSNR	Limited evidence for proton performance; no prospective workflow
Rossi and Cerveri (10)	Pelvis; 56 eligible patients	Public PRD cohort; device NR	CT reference; isocenter-based rigid alignment for supervised training/evaluation	Paired supervised versus unpaired comparison; four-fold validation	U-Net/CycleGAN; 2D	Internal; single public acquisition domain	No	<4 s/~70 slices (Tesla P100)	MAE, PSNR, SSIM	Image-only evaluation; single public acquisition domain
Wynne et al. (68)	Prostate/pelvis; 79 patients, 102 same-day image sets	One institution; device NR	Same-day QACT reference; evaluation registration NR	Unpaired CUT versus CycleGAN comparison	CUT/CycleGAN; 2D	Internal; one institution	No	NR; relative computational-cost comparison reported	MAE, RMSE, SSIM, FID	Internal image-only study; no locked external or dosimetric test
Li et al. (12)	Head and neck; 140 (training, 100; testing, 40)	One center; four linear accelerators	CT reference with a learned registration-noise model	Internal cross-device split	RegGAN; 2D	Internal cross-device testing; same institution	PTV/dose-difference analysis	NR	MAE, PSNR, SSIM	Devices from the same center; no prospective workflow
Peng et al. (13)	Brain, 41; head and neck, 47	One reported institution; device NR	Deformation-derived reference: DIR-transformed pCT target	Paired conditional training	cDDPM; 2D	Internal; one reported institution	No	~2 min/slice (reported hardware)	MAE, PSNR, NCC; same-data comparator	Internal registered targets; no dosimetric or workflow testing
Chen et al. (60)	Lung; 25 institutional patients plus selected public 4D-CBCT cases	One institution plus public cohort; institutional CBCT acquired with Varian TrueBeam	CT reference after two-stage deformable pairing	Paired L-DM; fine-tuned on the public cohort	L-DM; 2D	External adapted evaluation; public cohort used for fine-tuning; model not locked	No	~11 min/treatment fraction (two RTX 4070 Ti GPUs)	MAE, PSNR, NCC, SSIM	No dosimetric validation; external evidence confounded by fine-tuning
Hu et al. (16)	Head and neck, 30; thorax, 30 (80/20 split)	One tertiary hospital; GE CT and Elekta XVI CBCT	CT reference; ANTs affine registration for evaluation	Unpaired, internal anatomical-site split	IViT-CycleGAN; 2D	Internal cross-anatomical-site testing; same institution	Dose-distribution/DVH examples; quantitative acceptance criteria NR	NR	MAE, PSNR, SSIM; 3D reconstruction	Approximately six test patients per site; no independent center; limited dosimetric reporting
Huijben et al. (18)	CBCT task: brain, 270; pelvis, 270	Three Dutch centers; multiple acquisition settings	Challenge-defined paired CT/CBCT reference images	Heterogeneous submitted methods; held-out testing	Cross-paradigm benchmark; 2D/3D	Locked multicenter held-out testing	Photon and proton metrics	Submission-dependent; aggregated hardware-normalized time NR	Image similarity plus dosimetric ranking	Brain/pelvis benchmark only; no local workflow or prospective use
Hu et al. (57)	Brain, 105; head and neck, 108	One institution; GE CT and Elekta XVI CBCT	CT reference; ANTs registration	Paired internal training/test split	HUDiff; 2D	Internal; one institution	Dose-distribution and DVH examples	0.217s/image versus 0.007–0.009 s for GANs (eight V100 GPUs)	MAE, PSNR, SSIM, NCC	Single center; dosimetric examples without independent or workflow validation
Zhang et al. (15)	Pelvic development set plus external pelvic and head datasets; patient numbers NR	Three datasets/centers; device details NR	Paired reference images; registration details NR	Conditional diffusion; internal and external testing	Texture-preserving diffusion; spatial dimension NR	External image testing; model locking and adaptation status NR	No	NR	MAE, PSNR, SSIM	External image evidence only; sample/device details, dosimetry, and workflow time NR
He et al. (50)	Nasopharynx; 87 (training, 80; testing, 7)	One center; device NR	CT reference; registration method NR	Unpaired internal split	PE-CycleGAN; 2D	Internal; single-center	DVH and 3D γ	NR	MAE, PSNR, SSIM	Seven-patient test; no independent center or workflow validation
Hémon et al. (55)	Head and neck; 85 development/internal patients plus 3 OOD patients	One development center plus patients from an unseen center; device NR	CT reference; registration details NR	54/5/26 internal split plus three OOD cases	sCT and voxel-wise uncertainty models; spatial dimension NR	Limited external OOD testing; three unseen-center cases	DVH uncertainty in 16 internal test patients	NR	Voxel-wise error/uncertainty; case analyses	Very small external sample; no prospective QA workflow
Quintero et al. (65)	Abdomen; 65 (52/6/7)	One center; Philips Big-Bore CT; CBCT device NR	CT reference; rigid registration and normalized-intensity DIR compared in MIM	Internal split; three-fold development; repeated/MC uncertainty	U-Net, Bayesian U-Net, CycleGAN; 2D	Internal; single-center	2%/2 mm γ, PTV *D*_95_, liver *D*_mean_	NR	MAE, RMSE, SSIM, voxel-wise uncertainty	Seven-patient test; single-center; no workflow evaluation
Vestergaard et al. (9)	Head-and-neck proton therapy; 24 patients/35 CBCT scans	One center; imaging-system details NR here	Same-day reCT reference; clinical rigid registration plus method-specific DIR	Head-to-head evaluation; source of DL training data NR	Correction, two DIR methods, and DL sCT; 3D output	Internal; single-center	Proton range and clinical-plan DVH	Corrected CBCT <1 min; other methods NR	CT values, anatomical review, case outliers	Reference anatomy/position mismatch; single-center
Li et al. (17)	Thoracic phantom; patient sample size NR; 30 simulated motion scenarios	One center; Varian VitalBeam and CIRS dynamic thoracic phantom	Dynamic thoracic phantom; rigid registration and DIR with contour and plan propagation	Locked RegGAN-based workflow; training-cohort details NR	RegGAN workflow; 2D generation	Preclinical single-platform phantom evaluation; no external patient testing	Recalculation, film, and phantom γ	sCT approximately ~15 s; end-to-end 6–8 min	MAE, PSNR, SSIM	Single platform and mechanistic phantom; no prospective patient deployment

Sample sizes are reported at the patient level; time units and hardware information are retained as reported in the original studies. Reference-image and validation-independence terms are standardized from information reported in the original studies. NR indicates information that was not reported or could not be reliably extracted. Studies with non-conventional output targets are discussed in the text as methodological background and are not included as direct task evidence in this table.

### 3.6 Evidence maturity and clinical reliability

Evidence maturity and clinical reliability describe complementary aspects of validation. Evidence maturity reflects the independence, task relevance, and operational context of validation, whereas the four clinical-reliability dimensions identify which aspect of performance is evaluated. The two axes should therefore be considered jointly rather than combined into a single performance score. A study may provide relatively strong evidence in one dimension while remaining limited in others.

**Table 2** summarizes this two-axis framework. Validation maturity describes the context and independence of the supporting evidence, whereas the data and transferability, structural reliability, dosimetric validity, and deployment feasibility dimensions specify the clinical question addressed by that evidence.

**Table 2:** Two axis framework for evaluating evidence maturity and clinical reliability of sCT generation from CBCT.

Framework component	Definition or core question	Minimum evidence or recommended reporting	Interpretive boundary
Validation maturity			
Proof of concept	Does the method generate technically plausible sCT under the evaluated development conditions?	Internal data with image or structural endpoints demonstrating generation feasibility (13)	Demonstrates technical feasibility but does not establish dosimetric suitability, transferability, or workflow readiness
Internal validation	Does performance persist in patient-independent testing within the development institution or acquisition domain?	Independent internal test data with case-level image, structural, dosimetric, or uncertainty results (65, 68)	Supports performance within the sampled domain but does not establish independent external transferability
External validation	Does a locked model maintain performance outside the development domain?	Independent temporal, device, protocol, or institutional testing, or held-out multicenter evaluation, without adaptation to the test cohort (18, 55)	External evidence should be interpreted according to institutional independence, model locking, and any use of external data for adaptation
Preclinical task validation	Does the method support the intended treatment-related task under controlled patient or phantom conditions?	Clinical plans, appropriately acquired reference CT, modality-specific dosimetry, or dynamic-phantom testing (9, 17)	Supports task-specific performance but does not establish prospective operation in routine clinical practice
Workflow deployment evidence	Can a locked workflow operate prospectively or in shadow mode with predefined QA and recovery procedures?	End-to-end processing time, failures, retries, QA triggers, human review, override, and recovery records (17, 69)	Retrospective or phantom workflows cannot substitute for prospective evidence under the intended clinical conditions
Clinical-reliability dimensions			
Data and transferability (D)	Does model performance extend beyond the development data and registration pipeline?	Patient and center numbers, devices, acquisition protocols, data splits, registration procedures, model locking, adaptation status, and independent testing	Large samples or multi-device testing within one institution do not by themselves establish cross-center transferability
Structural reliability (S)	Does the generated sCT preserve patient-specific anatomy and dose-sensitive boundaries?	Regional HU errors, organ or lesion boundaries, Dice, HD95, expert assessment, uncertainty, and case-level failure analysis	Global image metrics may conceal localized structural errors, and reference-image mismatch can affect apparent performance
Dosimetric validity (O)	Can the generated sCT support the intended photon or proton dose-calculation task?	Patient-level dose-distribution comparison, DVH and target/OAR endpoints, and modality-specific proton RSP, WET, or range measures	Dosimetric evidence depends on treatment modality, anatomical site, dose engine, contours, plan, and reference conditions
Deployment feasibility (P)	Can the complete pipeline operate within the intended ART workflow in a timely and controllable manner?	Hardware, preprocessing, registration, inference, dose recalculation, QA, end-to-end time, failure and retry records, and human intervention	Model inference time alone does not represent complete workflow feasibility

Validation maturity describes the independence, task relevance, and operational context of the evidence, whereas the D/S/O/P dimensions describe the clinical-reliability domain evaluated. The two axes are complementary and are not intended to generate a composite performance score.

Supervised regression and adversarial generation have accumulated broader internal and site-specific dosimetric evidence, whereas registration-aware methods have undergone selected dosimetric and phantom evaluations. Diffusion-based studies remain concentrated mainly in retrospective proof-of-concept and internal-validation settings, with less evidence from independent task-specific or workflow evaluation (17, 18, 23, 24).

Evidence maturity should therefore be interpreted together with the specific clinical-reliability dimensions addressed by each study. Evidence in one dimension does not establish reliability in the remaining dimensions.

## 4 Clinical evaluation and translation

The clinical value of sCT generation from CBCT cannot be inferred solely from network architecture or global image-similarity metrics. Evaluation should also establish whether the generated images preserve patient-specific anatomy, support the intended dosimetric task, generalize beyond the development domain, and operate feasibly within the intended clinical workflow.

### 4.1 From image similarity to clinical task validation

#### 4.1.1 Image and structural evaluation

Existing studies commonly use mean absolute error (MAE), peak signal-to-noise ratio (PSNR), structural similarity index (SSIM), spatial nonuniformity (SNU), and normalized cross-correlation (NCC) to quantify agreement between sCT and reference CT (31). MAE quantifies the mean absolute HU difference, PSNR reflects global signal fidelity relative to the reconstruction error and image dynamic range, and SSIM measures similarity in local luminance, contrast, and structure. NCC and SNU characterize grayscale correspondence and spatial intensity uniformity, respectively (70).

These global metrics provide limited information about the tissue type and spatial distribution of errors. MAE does not distinguish errors occurring in bone, air, soft tissue, targets, or organs at risk (19). Similarly, PSNR, SSIM, NCC, and SNU may be insensitive to localized structural deformation, tissue-boundary displacement, or abnormal HU values in dose-sensitive regions. Image-based evaluation should therefore be complemented by region-specific HU errors, the Dice coefficient, the 95th-percentile Hausdorff distance (HD95), surface distance, contour displacement, expert assessment, and case-level failure analysis. Studies limited to image or structural endpoints support conclusions only about image or structural performance under the evaluated conditions and do not establish dosimetric validity or suitability for clinical dose calculation.

Structural evaluation also depends on the validity and spatial correspondence of the reference images. Residual misalignment between CBCT and reference CT may be attributed incorrectly to synthesis error, whereas excessive registration may obscure genuine interfractional anatomical changes (16, 65). Studies should therefore report the timing of reference CT acquisition, registration method and quality control, region-of-interest definitions, case-level error distributions, and outlier cases, while distinguishing reference-image mismatch from model-related synthesis error.

Uncertainty estimation may help identify high-error voxels or cases, but uncertainty is not itself a measure of accuracy. Its clinical utility depends on calibration, failure-detection performance, and integration with quality assurance (QA) triggers and human-override procedures (55, 65).

#### 4.1.2 Dosimetric evaluation

Improved image quality does not necessarily imply dosimetric reliability (65). Reduced noise, enhanced contrast, and lower global MAE primarily indicate greater image-level agreement between sCT and reference CT, whereas dose calculation is sensitive to local HU values, tissue boundaries, and density errors along the beam path (28). Errors near target boundaries, bone, air cavities, and soft-tissue interfaces may affect electron density estimation, dose recalculation, and treatment plan evaluation (9, 17).

Existing studies commonly use the *γ* index, gamma pass rate (GPR), and dose–volume histogram (DVH) to compare dose distributions calculated from sCT and reference CT (34). The *γ* index combines dose difference and distance to agreement criteria, with settings such as 3%/3 mm, 2%/2 mm, and 3%/2 mm reported in radiotherapy dose-comparison studies (71, 72). These settings are study-specific evaluation criteria and should not be interpreted as universal clinical acceptance thresholds for sCT. AAPM TG-218 addresses measurement-based patient-specific IMRT quality assurance rather than validation of sCT for dose calculation; its recommended criteria should therefore not be directly adopted as sCT acceptance thresholds (71). Studies using *γ* analysis should report the dose-difference and distance to agreement criteria, global or local normalization, low-dose threshold, evaluation region, and patient-level GPR distribution. A cohort-level mean GPR alone does not establish dosimetric validity.

DVHs provide clinically relevant summaries of target coverage and doses to organs at risk (OARs), thereby linking sCT generation to treatment plan evaluation (31, 34, 69, 73). In addition to complete DVH curves, studies should report target *D*_95_, *D*_98_, and *D*_2_ and relevant mean, maximum, or site-specific dose endpoints for OARs. These endpoints should be interpreted within the evaluated treatment plan, anatomical site, dose-calculation setting, and reference-image conditions rather than as universal acceptance thresholds for sCT.

Proton therapy is particularly sensitive to tissue relative stopping power, beam-path composition, and changes in air cavities. Proton evaluation should therefore include relative stopping power, range deviation, and water-equivalent thickness (WET) error in addition to clinically relevant dose endpoints (9, 25, 26). Photon and proton dose calculations differ in their sensitivity to local density and beam-path errors; evidence for the two modalities should therefore be evaluated using modality-specific endpoints and interpreted separately.

Evaluation should therefore distinguish global image similarity, regional quantitative and structural endpoints, and task-specific dosimetric endpoints, with each interpreted according to the evidence it directly supports (**Table 3**).

**Table 3:** Evaluation endpoints and interpretive boundaries for sCT generation from CBCT.

Endpoint	Evidence domain	Information provided	Interpretive boundary	Recommended reporting/use
MAE	Image/intensity	Mean absolute HU difference between sCT and reference CT	Does not identify the spatial distribution or tissue type of errors and does not establish structural or dosimetric reliability	Report patient-level distributions and interpret together with regional HU and structural endpoints
Region-specific MAE/HU error	Quantitative/HU	HU accuracy in bone, air, soft tissue, targets, and OARs	Depends on segmentation quality, anatomical correspondence, and reference-image registration	Report tissue- and region-specific errors, particularly in dose-sensitive structures
PSNR	Image/signal fidelity	Global signal fidelity relative to reconstruction error and image dynamic range	Insensitive to localized structural deformation and dose-sensitive boundaries	Use for image-level comparison under the same dataset and evaluation pipeline
SSIM	Image similarity	Similarity in local luminance, contrast, and structure	Does not directly quantify HU accuracy, electron density, or patient-specific anatomical-boundary preservation	Use as a supplementary image-similarity endpoint rather than evidence of dosimetric validity
NCC	Image/global consistency	Grayscale correspondence between sCT and reference CT	May conceal localized intensity, structural, and boundary errors	Use as a supplementary measure of global grayscale correspondence
SNU	Image/artifact-related	Spatial intensity nonuniformity within homogeneous regions	Does not assess anatomical-boundary preservation, HU accuracy across tissue classes, or dosimetric effects	Use to characterize correction of intensity nonuniformity and related CBCT artifacts
Dice, HD95, and surface distance	Structural/geometric	Overlap and boundary agreement of targets, OARs, or tissues	Depend on contour quality, reference-image correspondence, and registration	Report structure-specific and patient-level results to assess anatomical preservation
Uncertainty and failure-detection performance	Supporting reliability	Ability to identify high-error voxels, abnormal cases, or out-of-distribution inputs	Uncertainty is not a direct measure of image accuracy or dosimetric validity and depends on calibration and predefined failure criteria	Report calibration, sensitivity, specificity, error–uncertainty relationships, and predefined QA-triggering conditions
γ index	Dosimetric/spatial agreement	Joint assessment of dose difference and distance-to-agreement	Strongly depends on dose-difference and distance-to-agreement criteria, normalization, low-dose threshold, and evaluation region; no criterion constitutes a universal sCT acceptance threshold	Report all γ settings explicitly and interpret the result as a study-specific dose-comparison endpoint
GPR	Dosimetric/summary agreement	Proportion of evaluated points or voxels satisfying the specified γ criteria	Criterion-dependent and does not indicate the location or clinical relevance of dose differences; cohort-level means may conceal patient-level failures	Report patient-level distributions together with the complete γ analysis settings
DVH	Dosimetric/clinical planning	Dose–volume distributions of targets and OARs	Does not fully represent spatial dose errors or localized hot and cold spots and is dependent on contours and plan conditions	Report complete DVH comparisons together with relevant target and OAR dose endpoints
Target/OAR dose endpoints	Dosimetric/clinical planning	Target coverage, high-dose regions, and doses to critical structures	Depend on anatomical site, contours, treatment plan, dose-calculation settings, and reference images; no single endpoint provides a universal sCT acceptance threshold	Report patient-level differences for prespecified target and OAR endpoints relevant to the intended dose-calculation task
RSP, WET, and range error	Proton dosimetry	Differences in relative stopping power, water-equivalent path length, and proton range	Highly sensitive to beam-path composition, reference positioning, air cavities, and anatomical change; photon-oriented endpoints alone do not establish proton dosimetric validity	Report proton-specific quantitative endpoints together with clinically relevant plan-dose measures

Image and structural endpoints characterize image agreement and anatomical preservation, whereas dosimetric endpoints assess task specific dose calculation. Image only results do not establish dosimetric validity, and reported *γ* criteria should be interpreted as study specific comparison settings rather than universal sCT acceptance thresholds.

### 4.2 Clinical stratification by anatomical site and treatment modality

Validation requirements for sCT generation from CBCT differ by anatomical site and treatment modality. Anatomical sites vary in motion patterns, tissue interfaces, and artifact sources, whereas photon and proton therapy differ in their sensitivity to localized density errors. Evidence should therefore be interpreted within the relevant anatomical and treatment context rather than extrapolated directly across sites or modalities. **Table 4** summarizes the principal site- and modality-specific validation considerations.

**Table 4:** Site and modality specific validation considerations for sCT generation from CBCT.

Clinical stratum	Site- or modality-specific challenge	Critical sensitivity	Validation emphasis
Stratification by anatomical site			
Head and neck	Tumor regression, weight loss, swallowing, changes in shoulder position, and dental artifacts	Bone, teeth or metal, oral and sinonasal air cavities, and narrow target–OAR boundaries	Use same-day or otherwise appropriately justified reference CT where feasible; report HU errors in bone, air, and soft tissue, contour or surface errors, and case-level outliers; use photon DVH/γ or proton range/WET endpoints according to treatment modality (9, 12, 55)
Thorax	Respiratory and cardiac motion, tumor movement, changes in lung inflation, and field-of-view truncation	Air–lung–soft-tissue interfaces, steep density gradients, and respiratory-phase mismatch	Use phase- or breath-hold-matched reference images where applicable; report lung and tumor HU and boundary errors, dose results in relation to motion conditions, and dynamic-phantom or end-to-end evaluation when relevant (17, 46, 60)
Abdomen	Respiratory motion, peristalsis, low soft-tissue contrast, and variation in organ or gas filling	Bowel gas, hepatic soft tissue, moving target–OAR interfaces, and noncorresponding anatomy	Report regional HU values in liver, soft tissue, and gas, target and OAR boundary errors, task-specific dose endpoints, and case-level uncertainty or failure when available (31, 65)
Pelvis	Bladder and rectal filling, bowel gas, prostate or uterine displacement, hip prostheses, and field-of-view limitations	Bone–soft-tissue and gas interfaces, metal-related artifacts, and targets adjacent to bladder and rectum	Report tissue-specific HU and contour errors, failures associated with metal or field-of-view limitations, relevant bladder or rectal filling conditions, and patient-level photon or proton dosimetric endpoints (10, 18, 42)
Stratification by treatment modality			
Photon ART	Interfractional anatomical change may alter electron-density mapping and target/OAR dose	Local HU and boundary errors may affect dose distributions, target coverage, and OAR dose	Report the complete settings of study-specific γ analysis together with DVH and target/OAR endpoints; maintain consistent contours and dose-calculation settings for sCT and reference comparisons (18, 34, 65)
Proton ART	Range is sensitive to daily anatomy, tissue composition, and changes in air cavities	Errors in relative stopping power, WET, bone, and air along the beam path may alter distal range	Report RSP, WET, or range error together with clinically relevant plan-dose endpoints and beam-path analysis; use same-day reference CT where feasible and report case-level outliers (9, 25, 26)

#### 4.3.1 Patient-level failure detection and structural reliability

The clinical risks of sCT generation from CBCT are determined not only by cohort-level performance but also by localized structural and HU failures in individual patients. Aggregate metrics may conceal targetboundary displacement, hallucinated structures, abnormal air cavities, and errors in local tissue-density recovery (49, 65). Uncertainty-aware studies have examined associations between voxel-wise uncertainty and sCT or dose error (55, 65), but evidence remains limited regarding calibration, predefined failure definitions, and the ability of uncertainty or out-of-distribution measures to identify clinically relevant high-error cases.

#### 4.3.2 External generalization and prospective validation

Validation independence should be characterized by the relationship between the evaluation cohort and the development data rather than by a binary designation of external testing. Testing within the development institution and acquisition domain constitutes internal same-domain evaluation, whereas testing across devices, vendors, acquisition protocols, or temporal cohorts within the same institution provides internal cross-domain evidence. Evaluation on data from another institution after fine-tuning or domain adaptation should be reported as external adapted evaluation rather than independent external validation. Locked external validation requires evaluation of an unchanged model on an independent-center cohort without use of that cohort for model adaptation. Held-out multicenter testing provides broader independent evaluation, whereas very small unseen-center or out-of-distribution samples should be identified as limited OOD testing rather than full external validation (24, 51, 74, 75).

Reports of external validation should specify institutional independence, device and vendor differences, acquisition protocols, temporal independence, model locking, and any use of external data for finetuning or adaptation. The preprocessing and evaluation pipelines should also be reported, together with aggregate performance, performance degradation relative to the development domain, and case-level failures. Multicenter benchmarks improve cross-method comparability but do not replace site-, device-, and workflow-specific validation at the target institution (18).

Most existing studies remain retrospective, with limited prospective evidence and real-world operational records (18, 24). Before clinical deployment, prospective shadow-mode studies should record model outputs, human judgments, failure types, reasons for override, and recovery procedures without allowing model outputs to affect treatment decisions. Such studies should also prespecify the intended use, exclusion criteria, acceptance criteria, and model-specific quality-control procedures (69).

#### 4.3.3 Workflow efficiency and quality assurance

Model inference time represents only one component of the ART workflow. The complete process also includes data transfer, image preprocessing, registration, sCT postprocessing, contour propagation, dose calculation, plan evaluation, QA, and recovery from abnormal conditions (17, 68). Reported processing times are therefore not directly equivalent to end-to-end workflow performance.

Current workflow evidence remains limited with respect to failure and retry rates, human intervention, fallback procedures, and post-deployment monitoring (17, 69). Dynamic-phantom and shadow-mode studies can assess technical completeness, but they do not by themselves establish operational reliability during prospective patient use.

### 4.4 Future research directions

#### 4.4.1 Standardized evaluation and benchmarking

Multicenter public challenges such as SynthRAD provide a basis for standardized data partitioning and image- and dose-based evaluation (18, 74). Future benchmarks should harmonize reference-image definitions, registration procedures, dataset splits, dose-calculation settings, model-version reporting, and patient-level endpoints. Public benchmarks should be complemented by site-specific QA because benchmark performance does not by itself establish performance within a local clinical workflow.

Evaluation should retain modality-specific endpoints. Proton studies should report relative stopping power (RSP), water-equivalent thickness (WET), range-related measures, and relevant plan-dose endpoints rather than directly transferring criteria used for photon dose comparison (25, 26, 39).

#### 4.4.2 Risk-aware evaluation and quality assurance

Patient-level failure detection, uncertainty estimation, and out-of-distribution detection should be evaluated against predefined failure definitions and calibrated using independent data (55, 65). Their clinical use requires explicit relationships between risk estimates, QA triggers, human review, and fallback procedures.

Failure analysis should report whether high-error voxels or cases are detected before the generated sCT contributes to treatment decisions. Sensitivity, specificity, calibration, false-negative failures, and the consequences of triggered human review should be reported where applicable.

#### 4.4.3 Model optimization constrained by clinical endpoints

Accelerated diffusion, latent representations, model distillation, and lightweight networks may reduce inference latency, but computational speed and global image similarity should not be the sole optimization objectives (24, 67). Model comparisons should retain consistent regional HU, structural, dosimetric, uncertainty, and end-to-end timing endpoints.

Anatomical priors, target and OAR constraints, segmentation-assisted tasks, dose-consistency objectives, and uncertainty estimation may be incorporated as auxiliary constraints or multitask objectives (16, 47, 55, 65). Their value should be determined by improvements in patient-specific structural preservation, regional HU accuracy, or downstream dosimetric performance rather than by auxiliary-task performance alone.

## 5 Conclusion

For organizational purposes, this review discusses deep learning methods for generating sCT from CBCT within four non-mutually exclusive methodological categories: supervised regression, adversarial generation, registration-aware approaches, and diffusion-based generation.These paradigms differ in their paired-data requirements, structural constraints, computational demands, and supporting validation evidence. Current evidence does not establish that any single paradigm consistently outperforms the others across anatomical sites, imaging devices, and treatment modalities. Most studies remain at the proof-of-concept or single-center internal-validation stage, although selected studies have incorporated patient-level dosimetric evaluation, multicenter benchmarking, uncertainty analysis, and dynamic-phantom testing. Prospective operation, patient-level failure management, continuous QA, and integration into complete clinical workflows remain insufficiently evaluated.

This review assessed the available evidence across four dimensions: data and transferability, structural reliability, dosimetric validity, and deployment feasibility. These dimensions were considered together with validation maturity to identify the principal barriers to clinical translation. As a narrative review, this study did not use protocol-based dual-reviewer screening, formal risk-of-bias assessment, or quantitative synthesis. Although a structured literature search was used to improve transparency, study selection and evidence interpretation remain subject to incomplete study capture and interpretive judgment. The included studies were also heterogeneous in their data sources, devices, acquisition protocols, reference CT images, registration procedures, dose-calculation settings, and evaluation endpoints. Cross-study comparisons and broader generalization of the findings should therefore be interpreted cautiously. The proposed framework is intended as a narrative synthesis tool and should not be interpreted as a clinical acceptance criterion or regulatory standard.

Future studies should evaluate locked models across independent centers, devices, and acquisition protocols; report patient-level structural and HU errors; and use dosimetric endpoints appropriate to the anatomical site and treatment modality. Uncertainty estimation and anomaly detection should be linked to predefined QA thresholds, human review, and fallback procedures. End-to-end processing time, failure and retry rates, and recovery pathways should be evaluated using representative hardware and clinical workflows. sCT generation can be considered sufficiently supported for dose recalculation, plan evaluation, and treatment adaptation in ART only after structural reliability, dosimetric validity, risk-control capability, and deployment feasibility have been demonstrated within a clearly defined intended-use setting.
